# Fermentation quality of herbal tea residue and its application in fattening cattle under heat stress

**DOI:** 10.1186/s12917-021-03061-y

**Published:** 2021-11-12

**Authors:** Xiaona Zhuang, Zujing Chen, Xiaohong Sun, Fangjun Li, Junyi Luo, Ting Chen, Qianyun Xi, Yongliang Zhang, Jiajie Sun

**Affiliations:** Guangdong Provincial Key Laboratory of Animal Nutrition Control, Guangdong Engineering & Research Center for Woody Fodder Plants, National Engineering Research Center for Breeding Swine Industry, Guangdong Laboratory for Lingnan Modern Agriculture, South China Agricultural University, Guangzhou, 510642 Guangdong China

**Keywords:** Herbal tea residue, Microorganism, Fattening cattle, Fermented feed, Heat stress

## Abstract

**Background:**

Herbal tea residue (HTR) is generally considered to be the waste of herbal tea beverage production while it still retains rich nutrients and active substances. The main aim of the present study was to investigate the effect of fermentation technology on improving the quality of HTRs, and focus on the fermented HTR-induced alleviation of summer heat stress in fattening cattle.

**Results:**

In this study, the waste HTR was fermented and then fed to a total of 45 fattening cattle that were divided into 3 groups (fermented HTR replaced 0, 15, 30% of the forage component of the diet), and the feeding experiment was lasted for 40 days. The physiological indexes, growth performance and fecal microbiota of fattening cattle were evaluated and results showed that fermented HTR could effectively reduce the respiratory rate and rectal temperature of fattening cattle under heat stress, increase the daily feed intake and daily gain, and improve the antioxidant content and blood immune index. In addition, we studied the fecal microbiota composition of 6 fattening cattle in control and 30% HTR substitution groups and found fermented HTR significantly changed the composition of fecal microbiota and increased microbial diversity, and correlation analysis suggested that the bacteria were closely related to fecal SCFA levels of fattening cattle under heat stress.

**Conclusions:**

In this study, fermented HTR replaced 30% of the forage component of the diet that can change the intestine microorganisms, maintain health and alleviate the heat stress of fattening cattle.

**Supplementary Information:**

The online version contains supplementary material available at 10.1186/s12917-021-03061-y.

## Background

Herbal medicine has a long history of being used to prevent and treat diseases in China [1]. Studies have shown that drinking herbal tea can help to relieve heat in the body and the symptoms of sore throat caused by summer heat stress [2]. Herbal tea drinks are consumed widely because of their natural ingredients, convenient drinking, and unique health benefits [3–5]. In some areas, such as Guangdong, China, drinking herbal tea has even become part of the culture [6]. More and more people are consuming herbal tea drinks, requiring large scale tea production and resulting in large amounts of HTR [7]. In the past, most of tea residues were regarded as waste and were burned or dumped into landfill [8], which was not only environmentally damaging, but also a represented a waste of resources. Fortunately, researchers are now paying attention to the resource utilization of tea residues. Researchers have found that tea residues can significantly improve the soil fertility, leading to their use as organic fertilizers [9]. In addition, tea residues have been developed as a non-conventional water adsorbent to remove water pollutants like methylene blue [10], antibiotics [11], and heavy metals, such as chromium [12], cadmium [13], copper [14], lead, zinc [15], and arsenic [16] from aqueous solutions.

Alternatively, tea residues can be used as an unconventional feed resource in animal production. Generally, during tea production, not all of the effective substances are completely dissolved, thus some tea residues still retain a good proportion of the nutrients and bioactive compounds [17]. Like tea residues, it is reported that HTR contains many residual bioactive substances, such as phenols, polysaccharides, organic acids, alkaloids, and essential oils, which have been proven to have anti-oxidant and anti-bacterial effects [18]. Moreover, tea residues also contain protein, carbohydrate, fat, fiber, vitamins, and minerals [18]. Previous studies have used tea residues as substitutes for antibiotics to help improve the gut microbial composition of grower pigs [19]. In addition, tea residues improved the abdominal fat accumulation of broiler chickens, and had a positive impact on egg yolk quality [20]. Besides, researchers demonstrated the potential of tea residues for feeding ruminants [17]. In another study, a fermented herbal residue was fed to Holstein heifers, which effectively promoted the growth and immune performance of cows under heat stress [21].

In our paper, the tested HTRs included *Plumeria rubra* flowers, Honeysuckle flowers, Chrysanthemum flowers, Mesona chinensis leaves, *Prunella vulgaris* leaves, Microcos paniculata leaves and Liquiritia glycyrrhiza roots. The potential feeding value of the HTRs prompted us to develop a suitable technology for animal production. Currently, to apply HTRs, problems of storage, unbalanced nutrition, and poor palatability need to be solved. Therefore, the main aim of the present study was to investigate the effect of fermentation technology on improving the quality of HTRs, and focus on the fermented HTR-induced alleviation of summer heat stress in fattening cattle.

## Results

### Nutritional components and changes to the HTR after fermentation

As shown in Table S1, the HTR contained a high water content (75.10%) and the dry matter content was 24.90%. As a proportion of the dry matter content, the crude protein, crude fat, and ash contents were 13.10, 2.60, 6.69%, respectively. In addition, HTR also contained minerals, and the contents of potassium, calcium, magnesium, phosphorus, sulfur, and chlorine were 0.61, 1.14, 0.40, 0.25, 0.21, and 0.51%, respectively. The acid detergent fiber (ADF) and neutral detergent fiber (NDF) were 39.8 and 54.3%; contents of lactate acid, acetic acid, and butyric acid were 4.67, 1.58, and 0.35%, respectively; and the water soluble carbohydrate (WSC) was 3.10%. The net energy for maintenance and net energy gain of HTR were 0.79 and 0.25 Mcal/kg DM, respectively.

As shown in Table 1, the pH value of the mixture of the HTR, oat hay, and bacteria decreased from 4.95 to 3.89 after fermentation. On the basis of dry matter, the crude protein in the HTR increased by 1.07% compared with that of the unfermented HTR. The contents of NDF and ADF decreased by 5.82 and 4.32% respectively. On the basis of dry matter, the contents of lactate, acetic acid, and propionic acid increased to 6.93, 0.62 and 112.33 g/kg DM, respectively. Basal diet composition and nutritional level of bovine feeding were shown in Table S2. Compared with CN group, LC group and HC group had more abundant crude protein (11.25, 11.32 and 11.35%, respectively). In addition, compared with CN group, LC group and HC group also had more abundant crude fat (3.15, 3.2 and 3.22%, respectively), calcium (0.43, 0.65 and 0.79%, respectively) and phosphorus (0.45, 0.53 and 0.48%, respectively).

**Table 1:** Nutrient composition of fermented herbal tea residue for 0 and 30 days

Item	Fermentation for 0 days	Fermentation for 30 days
PH	4.95 ± 0.03	3.89 ± 0.04
Dry matter (%)	26.58 ± 0.74	24.94 ± 0.51
Crude protein (%)	8.85 ± 0.98	9.92 ± 0.82
Ash (%)	6.17 ± 0.04	6.56 ± 0.06
Neutral detergent fiber (%)	60.11 ± 1.30	54.29 ± 0.86
Acid detergent fiber (%)	34.33 ± 0.28	30.01 ± 0.36
Acetic acid (g/kg)	1.04 ± 0.01	6.93 ± 0.06
Propionic acid (g/kg)	0.02 ± 0.004	0.62 ± 0.009
Lactic acid (g/kg)	15.74 ± 0.002	112.33 ± 0.004
NEmf (MJ/kg)	5.68 ± 0.09	5.76 ± 0.11

The values were calculated as the means ± standard error of the mean (*N* = 6), except for dry matter, others item are based on dry matter

### Temperature and humidity index in the cowshed

The daily changes in ambient temperature and THI among the three time points is shown in Fig. S1. In general, the THI classification of heat stress is as follows: No heat stress when THI ≤ 72; mild heat stress when 72 < THI ≤ 79; high heat stress when 79 < THI ≤ 84; and severe heat stress when THI > 84 [22]. During the experiment, the mean THI of the barn was 81 (range 79-84), 86 (more than 84), and 79 (range 72-79) in the morning (08:00), afternoon (15:00), and evening (22:00), respectively. The total average THI was 82, which was between 79 < THI < 84. Therefore, we concluded that fattening cattle were in a high heat stress state during the whole period of the formal experiment.

### Effects of fermented HTR on RR and RT of fattening cattle under heat stress

The daily average RR of CN group was 87.04 breaths/min, and RT was 38.84 °C; RR and RT of LC group were 83.39 breaths/min and 38.75 °C, respectively; and of the HC group they were 80.23 breaths/min and 38.63 °C (Table 2). At three measurement times, the RR and RT of LC had no significant differences with CN group, while HC group showed significantly reduced RR at 15:00 and 22:00 (*P* < 0.05) and a reduced RT at 8:00 and 15:00 (*P* < 0.05) compared with those of the CN group. At 15:00, the RT of the HC group was significantly lower than that of the LC group (*P* < 0.05). Therefore, 30% fermented HTR replacement could effectively alleviate the RR and RT of fattening cattle under heat stress.

**Table 2:** Effects of fermented herbal tea residue feed on respiratory rate and rectal temperature of fattening cattle

Items	Time	CN	LC	HC
Respiratory rates (breaths/min)	8:00	87.12 ± 1.25	86.67 ± 1.12	85.00 ± 1.48
	15:00	87.50 ± 2.80^a^	83.00 ± 2.80^ab^	78.50 ± 1.00^b^
	22:00	86.50 ± 3.20^a^	80.50 ± 3.43^ab^	77.20 ± 1.74^b^
Rectal temperature (°C)	8:00	38.85 ± 0.04^a^	38.75 ± 0.04^ab^	38.61 ± 0.05^b^
	15:00	38.88 ± 0.06^a^	38.78 ± 0.05^ab^	38.60 ± 0.08^c^
	22:00	38.80 ± 0.05	38.73 ± 0.07	38.68 ± 0.07

The values were showed as the means ± standard error (*N* = 15); Different letters showed significant difference (*P* < 0.05), while the same letter or no letter showed no significant difference (*P* > 0.05)

*HTR* herbal tea residue, *CN* no herbal tea residues, the control group, *LC* 15% fermented HTRs replaced, the 15% replacement group, *HC* 30% fermented HTRs replaced, the 30% replacement group

### Effects of fermented HTR on feed intake and weight gain

Compared with the CN group, the feed intake and daily gain of the LC and HC increased gradually, which correlated positively with the increase in fermented HTR replacement (Table 3). The feed intake and average daily gain of the HC group were significantly higher than those of the CN group (*P* < 0.05). The average daily gain between the CN and LC groups showed no significant differences (*P* > 0.05); however, the feed intake increased significantly in the LC group (*P* < 0.05). In addition, fermented HTR also reduced the F/G value of fattening cattle significantly (*P* < 0.05). Therefore, fermented HTR could improve the feed intake and increased the feed conversion rate of fattening cattle under heat stress, and the effect of 30% fermented HTR replacement was better than that of 15%.

**Table 3:** Effect of fermented herbal tea residue feed on feed intake and weight gain of fattening cattle

Items	CN	LC	HC
Initial weight (kg)	518.29 ± 27.24	521.43 ± 38.45	524 ± 34.21
Final weight (kg)	542.94 ± 26.52	550.13 ± 37.35	558.33 ± 32.55
ADG (kg/day)	0.94 ± 0.05^b^	1.09 ± 0.09^ab^	1.26 ± 0.05^a^
Feed intake (kg/day)	12.64 ± 0.21^c^	13.41 ± 0.21^b^	14.27 ± 0.18^a^
Feed conversion ratio (F/G)	13.85 ± 1.07^a^	12.61 ± 0.53^b^	11.64 ± 0.40^c^

The values were calculated as the means ± standard error (*N* = 15); Different letters showed significant difference (*P* < 0.05), while the same letter or no letter showed no significant difference (*P* > 0.05)

*HTR* herbal tea residue; *CN* no herbal tea residues, the control group, *LC* 15% fermented HTRs replaced, the 15% replacement group, *HC* 30% fermented HTRs replaced, the 30% replacement group

### Effects of fermented HTR on serum biochemical indexes

Compared with the fattening cattle fed with a basal diet, the levels of HSP70 and LDH in the LC and HC groups decreased with the increase in fermented HTR substitution (*P* < 0.05), while IL-2 level increased gradually (*P* < 0.05). Compared with that in the CN group, the level of Cor in HC group reduced significantly in serum under heat stress (*P* < 0.05), and the levels of IgG and SOD significantly increased (*P* < 0.05). Compared with that in the CN group, the LDH level in the LC group decreased significantly (*P* < 0.05), and T-AOC level increased significantly in the LC group (*P* < 0.05). IgA, IL-6, ALT, AST, MDA and T4 levels had no significant among three treatments; IgA, IL-6, ALT and AST showed an increased trend, while MDA and T4 showed a decreased trend with the increase of fermented HTR substitution (Table 4). The above results showed that fermented HTR could improve the anti-heat stress and antioxidant capacity of fattening cattle, and the effect of 30% replacement was more obvious than that of 15% replacement.

**Table 4:** Analysis of serum indices in beef cattle under heat stressed between three groups

Items	CN	LC	HC
HSP70 (ng/mL)	18.67 ± 1.17^a^	17.78 ± 0.54^b^	16.87 ± 1.60^c^
Cor (ng/mL)	91.55 ± 1.83^a^	80.96 ± 0.60^ab^	72.63 ± 4.22^b^
LDH (U/L)	696.00 ± 10.65^a^	601.00 ± 8.97^b^	583.00 ± 3.76^b^
IgG (ng/mL)	7.96 ± 1.16^b^	9.62 ± 0.44^ab^	12.30 ± 1.18^a^
IgA (ng/mL)	3.57 ± 0.24	4.29 ± 0.43	4.60 ± 0.66
IL-2 (pg/mL)	308.75 ± 2.40^c^	332.81 ± 2.16^b^	340.47 ± 0.43^a^
IL-6 (pg/mL)	316.17 ± 15.25	327.69 ± 4.43	336.81 ± 4.80
ALT (U/L)	27.50 ± 2.02	29.50 ± 0.87	31.00 ± 3.21
AST (U/L)	78.00 ± 6.06	84.50 ± 9.49	90.25 ± 4.94
T-AOC (mmol/L)	0.27 ± 0.03^b^	0.36 ± 0.01^a^	0.33 ± 0.01^ab^
MDA (mmol/L)	25.21 ± 1.67	24.93 ± 0.00	23.47 ± 0.85
SOD (U/mL)	48.73 ± 2.79^b^	51.76 ± 0.58^ab^	56.43 ± 0.20^a^
T4 (ng/mL)	33.02 ± 1.06	33.47 ± 0.36	32.46 ± 0.70

The values were showed as the means±standard error (*N* = 6); Different letters showed significant difference (*P* < 0.05), while the same letter or no letter showed no significant difference (*P* > 0.05)

*Cor* Cortisol, *LDH* Lactate dehydrogenase, *IgA* Immunoglobulin A, *IgG* Immunoglobulin G, *IL-2* Interleukin-2, *IL-6* Interleukin-6, *ALT* Alanine aminotransferase, *AST* Aspartate aminotransferase, *T-AOC* Total antioxidant capacity, *MDA* Malondialdehyde, *SOD* Superoxide dismutase, *T4* Thyroxine, *HTR* Herbal tea residue, *CN* no herbal tea residues, the control group, *LC* 15% fermented HTRs replaced, the 15% replacement group, *HC* 30% fermented HTRs replaced, the 30% replacement group

Compared with that in the CN group, 30% fermented HTR could reduce blood glucose significantly, and 15 and 30% fermented HTR replacement could increase the content of total protein and albumin significantly. The content of blood urea nitrogen (BUN), total cholesterol (T-CHO) and triglyceride (TG) had no significant among three treatments; BUN and T-CHO showed a decreased trend with the increase of fermented HTR replacement, while TG showed an increased trend (Table 5).

**Table 5:** Effect of fermented herbal tea residue on biochemical indicators of fattening cattle during summer heat stressed

Items	CN	LC	HC
Glu (mmol/L)	2.09 ± 0.008^a^	2.06 ± 0.03^ab^	1.99 ± 0.006^b^
TP (g/L)	67.27 ± 2.15^b^	74.87 ± 0.67^a^	77.25 ± 1.01^a^
ALB (g/L)	32.23 ± 0.65^b^	33.67 ± 0.52^a^	36.65 ± 1.88^a^
BUN (mmol/L)	3.35 ± 0.31	3.04 ± 0.17610	2.69 ± 0.07
T-CHO (mmol/L)	2.72 ± 0.20	2.34 ± 0.28	2.46 ± 0.02
TG (mmol/L)	0.127 ± 0.03	0.14 ± 0.01	0.15 ± 0.009

The values were showed as the means ± standard error (*N* = 6); Different letters showed significant difference (*P* < 0.05), while the same letter or no letter showed no significant difference (*P* > 0.05)

*Glu* Glucose, *ALB* Albumin, *TP* Total protein, *BUN* blood urea nitrogen, *T-CHO* Total cholesterol, *TG* Triglyceride, *HTR* Herbal tea residue, *CN* no herbal tea residues, the control group, *LC* 15% fermented HTRs replaced, the 15% replacement group, *HC* 30% fermented HTRs replaced, the 30% replacement group

### 16S rRNA gene sequencing and annotation analysis

**Fig. 1:**
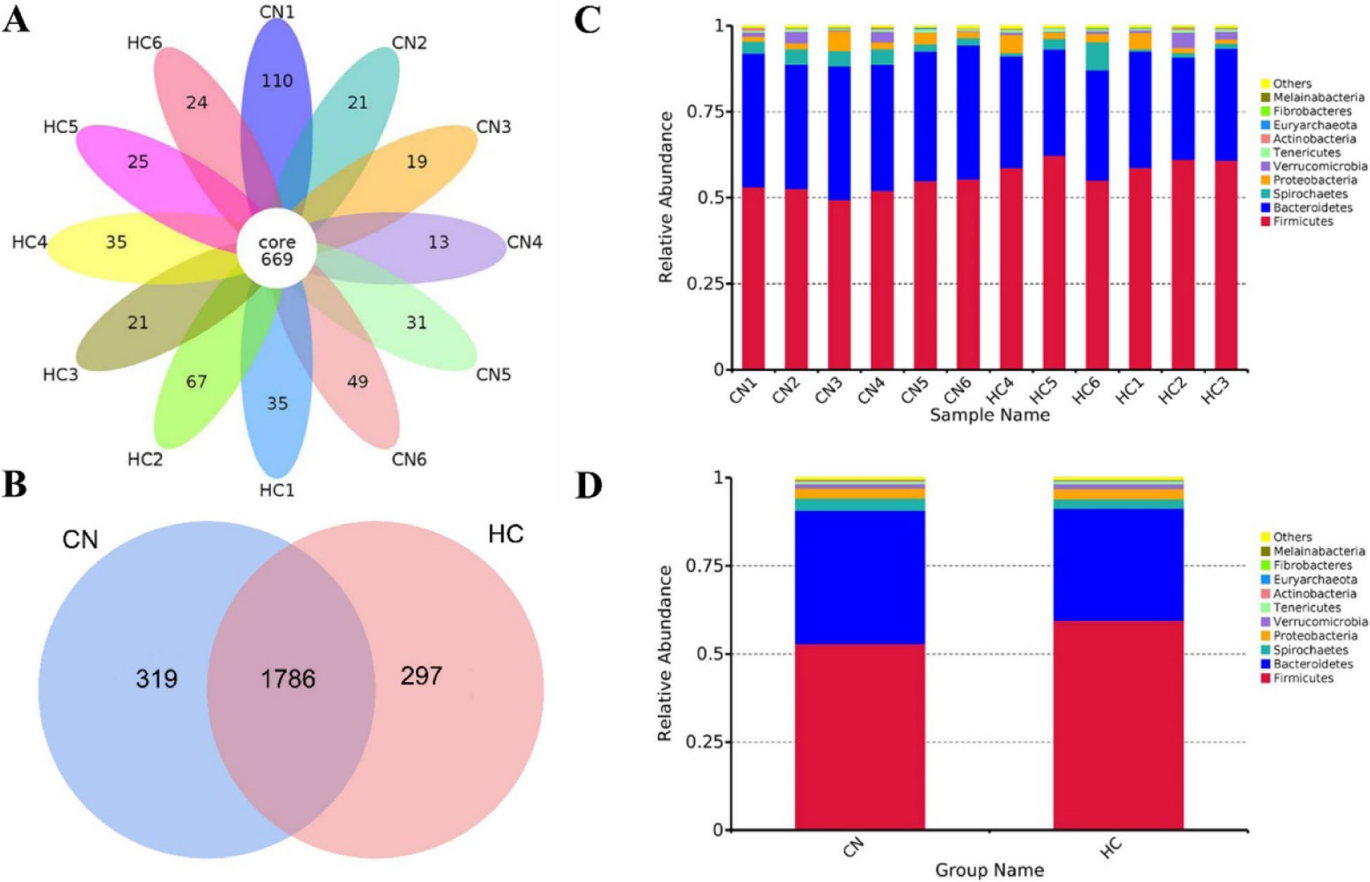
Common and specific OTU distribution of the fecal microbiota in each sample (**A**) and two experimental groups (**B**); relative abundance of fecal microbiota at the phylum level in each sample (**C**) and in two group (**D**). Note: OTU, operational taxonomic units; CN, no herbal tea residues, the control group; HC, 30% fermented HTRs replaced, the 30% replacement group

To study the effect of fermented HTR on intestinal microorganisms, we studied the fecal microbiota composition of 6 fattening cattle in control and 30% HTR replacement groups. We extracted the genomic DNA of 12 fecal samples from CN group and HC groups, and amplified the corresponding 16S DNA V3-V4 fragment. After sequencing, the CN group produced an average of 89,720 raw reads and the HC group produced an average of 90,069 raw reads. After splicing, the CN group produced an average of 88,745 combined reads and the average combined percentage was 98.92%. The HC group produced an average of 88,679 combined reads and the average combined percentage was 98.47%. After filtering low quality and short length fragments, the number of tag sequences for subsequent analysis were 58,016 nochime reads in the CN group, with an average effective rate of 64.93%, and 58,636 nochime reads in the HC group, with an average effective rate of 65.21% (Table S3A). The CN group data generated 1451 OTUs and the HC group generated 1446 OTUs on average (Table S3B). There were 669 core OTUs in the 12 samples (Fig. 1A); with 319 unique OTUs in the CN group and 297 OTUs in the HC group, while there were 1786 shared OTUs between the two groups (Fig. 1B; Table S3C).

The relative abundance of the top 10 fecal microbiota at the phylum level is shown Fig. 1 C and D. The most abundant bacteria at the phylum level were *Firmicutes* and *Bacteroidetes* within and between the CN and HC groups. The average percentage of *Firmicutes* in HC group was 59.54%, which was significantly higher than that in the CN group (52.94%) (*P* < 0.01). The average percentage of *Bacteroidetes* in the HC group was 31.87%, which was significantly lower than that in the CN group (37.87%) (*P* < 0.01). The annotated counts and percentages at the class, order, family, genus, and species levels are show in Table S4 and S5.

**Fig. 2:**
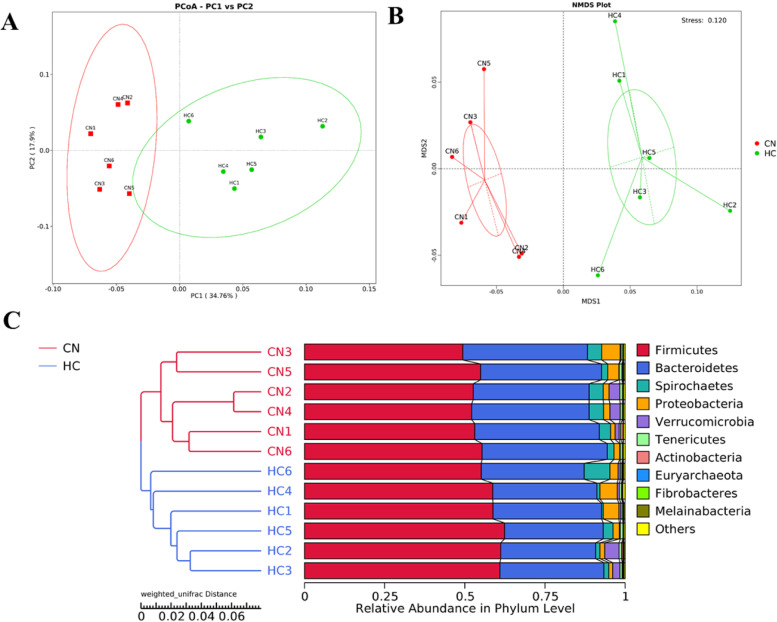
Principal component analysis between different groups. Note: PCoA (**A**), NMDS (**B**) and UPGMA (**C**) clustering representing for Principal Co-ordinates Analysis, Non-Metric Multi-Dimensional Scaling and Unweighted Pair-group Method with Arithmetic Mean algorithms, respectively; CN, the control group; HC is the 30% replacement group; OTU, operational taxonomic units

Observed species, the Shannon index, the Simpson index, Chao1, ACE, Good’s coverage, and PD whole tree reflected the richness and diversity of species in the samples. The species richness of the HC group was slightly higher than that of the CN group, but there was no significant difference (*P* > 0.05) (Table 6). The PCA between the groups is shown in Fig. 2A. We found that CN and HC groups samples were separated from each other, which reflected the influence of HTR substitution on microbial community changes. Similar results were obtained and showed in Fig. 2B. The UPGMA clustering tree was shown in Fig. 2C, which confirmed the significant structural separation of the fecal microflora between the two groups by measuring the similarity of microbial communities according to the degree of their overlap.

**Table 6:** Statistical analysis of sample complexity

Item	CN	HC	***P*** vaule
Observed species	1260.50 ± 15.1805	1265.50 ± 16.5726	0.828
Shannon	8.02 ± 0.0537	8.07 ± 0.0668	0.564
Simpson	0.99 ± 0.0006	0.99 ± 0.0010	0.568
Chao1	1316.69 ± 16.97	1319.46 ± 17.83	0.913
Ace	1321.61 ± 18.36	1320.44 ± 17.79	0.964
Goods coverage	0.99 ± 0.0001	0.99 ± 0.0001	0.334
PD whole tree	85.54 ± 2.3358	93.59 ± 2.7638	0.05

Alpha diversity reflected the richness and diversity of species in samples, and it has a variety of indicators: observed species, shannon, simpson, chao1, ace, goods coverage, PD whole tree. The higher the value of Observed species, the higher the species richness are. Shannon index evaluates the richness and evenness of species composition in the sample. The greater the value, the more abundant and evenly distributed the species are. The Chao1 and ACE indices measure species richness. Goods coverage index represents sequencing depth. PD whole tree index reflects phylogenetic diversity

*HTR* herbal tea residue, *CN* no herbal tea residues, the control group, *HC* 30% fermented HTRs replaced, the 30% replacement group

**Fig. 3:**
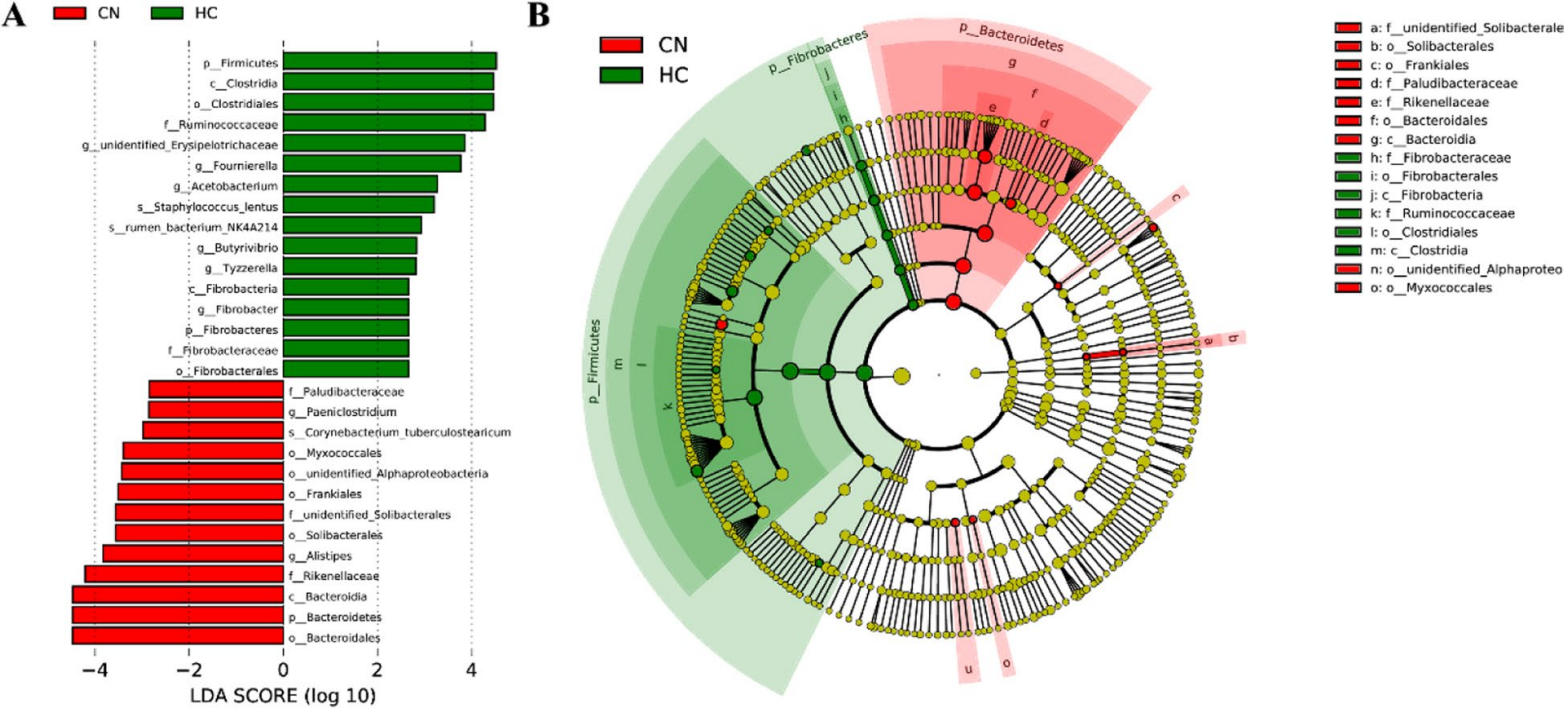
Taxonomic differences of fecal microbiota between CN and HC groups derived from the LefSe method. Note: **A** LDA scores observed for individual taxa that passed the LefSe significance threshold of 4. **B** Taxonomic cladogram. Taxa with enriched levels in CN were showed in red, whereas those with enriched levels in HC were showed in green. The brightness of the respective colors is proportional to the observed effect size. CN, no herbal tea residues, the control group; HC, 30% fermented HTRs replaced, the 30% replacement group

The data in Table S6 show that the levels the *Bacteroidia* and *Clostridia* in the HC group were significantly lower and higher, respectively, than those in the CN group at the class level (*P* < 0.01), while the *Bacteroidales* and *Clostridiales* (*P* < 0.01) showed the same trend at the order level. At the family level, the *Rikenellaceae* and *Paludibacteraceae* levels were decreased in the HC group (*P* < 0.01 and *P* < 0.05), and the *Ruminococcaceae* levels were increased (*P* < 0.05). At the genus level, *Erysipelotrichaceae* levels in the HC group increased (*P* < 0.01), and *Fournierella*, *Acetobacterium*, *Anaerovorax*, *Butyrivibrio*, and *Oscillibacter* levels in the HC group were also higher than those in the CN group (*P* < 0.05), whereas, the *Alistipes* levels were lower (*P* < 0.05). At the species level, the *rumen_bacterium_NK4A214* were enriched in the HC group (*P* < 0.05). LEFSe identified 29 differentially abundant taxonomic clades in the CN and HC groups, whose LDA scores were greater than 2.0 (Fig. 3). The results showed that at the phylum level, the *Bacteroidetes* were enriched in the CN group, while the *Fibrobacteres* and *Firmicutes* were enriched in the HC group.

### Fecal SCFAs concentration and their correlation with microbiota genera

**Fig. 4:**
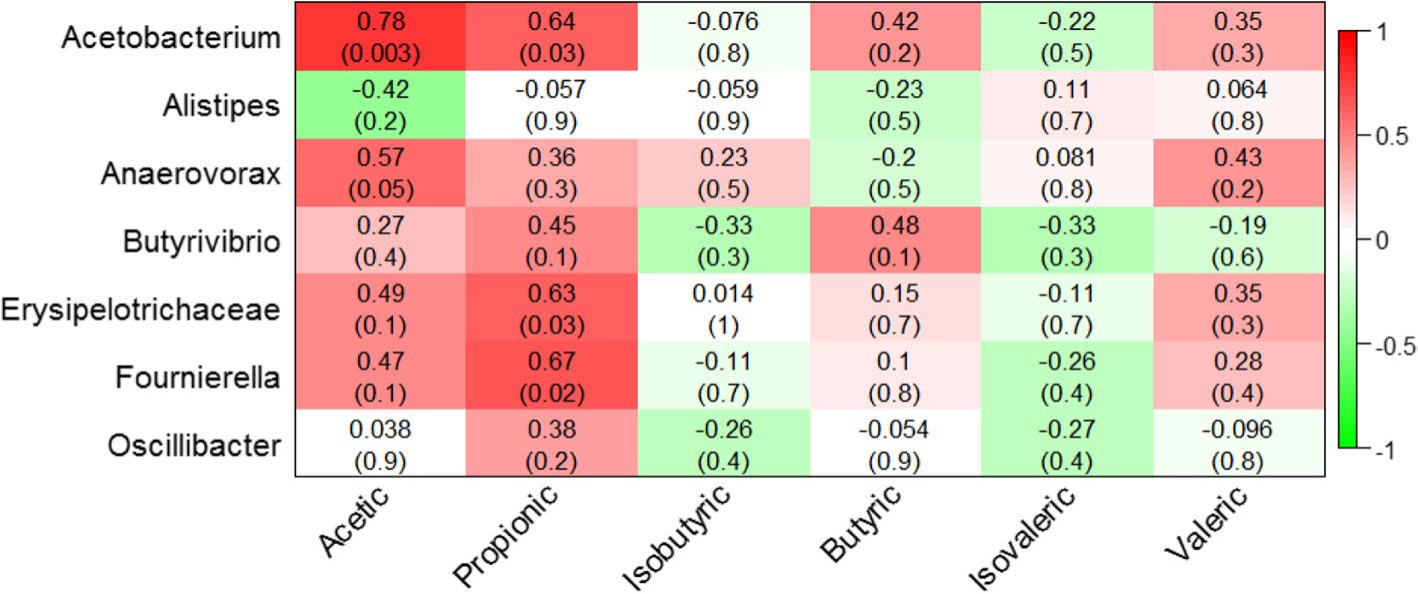
Statistical analysis of correlation between genus taxa and fecal SCFA. Note: Each cell contained the corresponding correlation and *P* value. The table is color-coded by correlation according to the color legend (Red indicates positive correlation and green indicates negative correlation)

SCFAs are the main products of microbial metabolism in the intestines, and the types and quantity of SCFAs are regulated by microbial species, diet, and the environment [23]. As shown in Table 7, the SCFAs of the fecal microorganisms in the CN, LC and HC groups showed different trends. The concentration of Aa increased gradually with the increase in fermented HTR substitution, at 13.33, 13.37, and 14.82 mg/g in the three groups, respectively. The Aa content in the HC group was significantly higher than that in the CN and LC groups. The concentrations of Pa (3.29, 3.46, and 3.542 mg/g, respectively), Ba (2.35, 2.51, and 2.49 mg/g, respectively), and Va (0.09, 0.09, and 0.10 mg/g, respectively) increased in the fermented HTR replacement groups, without statistical significance among the groups. The concentrations of Ia were 0.15, 0.13, and 0.17 mg/g, respectively, with the concentrations in HC group being significantly higher than those in the CN and LC groups. However, the concentration of Iva (0.22, 0.18, and 0.18 mg/g, respectively) in the LC and HC groups were significantly lower than those in the CN group. We also studied the correlation between SCFAs and significantly enriched fecal microbiota at the genus level. As shown in Fig. 4, there was a significant positive correlation between Aa and the relative abundance of *Acetobacterium* (*P* < 0.01), and a positive correlation with the relative abundance of *Anaerovorax* (*P* = 0.05). In addition, there was a positive correlation between Pa and the relative abundance of *Acetobacterium* (*P* < 0.05), *Erysipelotrichaceae* (*P* < 0.05), and *Fournierella* (*P* < 0.05). However, there was no significant relationship between Ia, Ba, Iva, Va, and *Acetobacterium*, *Alistipes*, *Anaerovorax*, *Butyrivibrio*, *Erysipelotrichaceae*, *Fournierella*, or *Oscillibacter* (*P* > 0.05).

**Table 7:** Effect of fermented herbal tea residue on the content of short chain fatty acids (SCFAs) in feces of fattening cattle (mg/g) (*N* = 6)

Item	CN	LC	HC
Acetic acid	13.33 ± 0.91^b^	13.37 ± 1.27^b^	14.82 ± 0.95^a^
Propionic acid	3.29 ± 0.22	3.46 ± 0.63	3.54 ± 0.26
Isobutyric acid	0.15 ± 0.01^b^	0.13 ± 0.01^b^	0.17 ± 0.02^a^
Butyric acid	2.35 ± 0.16	2.51 ± 0.22	2.49 ± 0.21
Isovaleric acid	0.22 ± 0.03^a^	0.18 ± 0.02^b^	0.18 ± 0.02^b^
Valeric acid	0.09 ± 0.01	0.09 ± 0.01	0.10 ± 0.02

The values were showed as the means±standard error (*N* = 6); Different letters showed significant difference (*P* < 0.05), while the same letter or no letter showed no significant difference (*P* > 0.05)

*CN* no herbal tea residues, the control group, *LC* 15% fermented HTRs replaced, the 15% replacement group, *HC* 30% fermented HTRs replaced, the 30% replacement group

## Discussion

HTR is the residual water-insoluble substance produced by herbal tea beverage, which has the potential to be used as feed [21]. Like corn silage with a high NDF and Lingnin contents [17], in this experiment, HTR contained more NDF (54.30% DM) and Lingnin (8.60% DM). Higher fiber level can enhance rumen peristalsis, and more NDF can also provide substrate and energy for roughage fermentation in rumen [24], indicating that HTR could be used as a source of ruminant roughage. To improve the palatability of HTR and solve the problem of storage, we fermented the HTR. However, the content of NDF was higher than 54% and the content of water soluble carbohydrate (WSC) was only 3.10%, which was not conducive to fermentation alone [25]. The addition of molasses increased the WSC of fermented materials [26], and oat hay helped to control the total water content at the beginning of fermentation. In addition, complex bacteria were added to help fermentation. Previous studies have shown that *Bacillus subtilis* grows strongly and can inhibit the growth of other aerobic and harmful microorganisms during the fermentation process [27]. At the same time, it can produce rich metabolites, such as organic acids and bioactive substances and during fermentation, *Bacillus subtilis* also produces a large number of enzymes [27]. Yeast is suitable for growth in acidic and humid environments containing sugar. Yeast can use monosaccharides or oligosaccharides produced in fermentation to produce alcohol and carbon dioxide, which is one of the best combination fermentation strains. *Lactobacillus* can inhibit the growth of spoilage bacteria, reduce the pH, maintain an acidic environment, and ensure the fermentation quality [28]. After fermentation, the content of lactic acid, acetic acid, and propionic acid increased, and the pH decreased to 3.89, which effectively inhibited the reproduction of undesirable bacteria, increased the content of crude protein and decreased the content of NDF, indicating a better fermentation quality. Previous study found that green tea residue silage could be used as a protein source for lactating dairy cows to replace part of the alfalfa and soybean meal in their diet, which had no adverse effect on the performance of the dairy cows [29], suggesting that fermented HTR could be used as feed resource.

In the subtropical region of southern China, the climate is hot in summer; therefore, beef cattle are vulnerable to summer heat stress, which affects their eating and health [30]. This results in growth retardation, which affects the development of the beef cattle industry [31]. It has been reported that heat stress can reduce the intake of feed and affect the metabolism after absorption [32]. The temperature and humidity index reflects the heat stress intensity of animals by measuring the temperature and humidity index of the breeding environment [33]. Our results showed that the total average THI was 82, which was between 79 < THI < 84. Therefore, we concluded that the fattening cattle were in a high heat stress state during the whole period of the formal experiment. RR and RT are indices used to quantify the physiological changes of animals under heat stress [34]. Replacement with fermented HTR was beneficial to reduce the RT and RR of fattening cattle in the heat stress environment, with the 30% fermented HTR having a more significant effect. This suggested that fermented HTR could alleviate heat stress symptoms of fattening cattle in summer, which is consistent with the results of other studies [35–37]. Chen found that Chinese herbal medicine could reduce significantly the RR of beef cattle under heat stress [36]. Song found that Chinese herbal medicine could reduce the RT for a certain period of time under high temperature [35]. When animals are exposed to heat stress, they eat less to reduce their heat production [37], which ultimately affects their growth and fattening.

Cor is a hormone secreted by the adrenal gland during stress. When the body is in the state of heat stress, it will stimulate the adrenal cortex to secrete Cor to adapt to the adverse environment [38]. Our results showed that in the heat stress environment, with the increase in fermented HTR in the diet, the amount of Cor secreted by the fattening cattle decreased gradually, indicating that fermented HTR could effectively alleviate the heat stress response of fattening cattle. This was consistent with the results of Song et al., who found that Chinese herbal medicine could reduce the secretion of Cor in beef cattle under heat stress [35] and this might be explained by the fact that herbal medicine active substances are retained in the HTR, which can relieve heat stress in summer. LDH participates in glycolysis. Hocking et al. found that high heat treatment can increase LDH activity [39]. In our results, LDH level decreased with the increase of fermented HTR replacement, indicating that fermented HTR could alleviate heat stress in fattening cattle.

When the animals are in the state of heat stress for a long time, the body’s immunity will decline [40]. IgG is the most abundant immunoglobulin in animal organs, which can protect the body from infection, and is an important immune index under heat stress [41]. Interleukin-2 (IL-2) is a cell growth factor in the immune system of the body, and is also an important indicator of cellular immune function under heat stress [42]. Furthermore, IL-6 is a proinflammatory cytokine that is associated with an increased inflammatory response [43]. Studies have shown that HTR can enhance the immune function of the human body [44]. In the present study, we found that the levels of IgG and IL-2 in the serum of beef cattle fed with fermented HTR increased significantly, which indicated that fermented HTR could enhance the immune function of beef cattle under heat stress.

The content of MDA indirectly reflects the degree of lipid peroxidation in tissues and cells, and can be used as a surrogate for the degree of cell damage [45]. SOD is an antioxidant enzyme that can scavenge free radicals in vivo. Studies show that heat stress can inhibit the expression of SOD [46]. T-AOC represents the antioxidant capacity of the whole body. In this study, the MDA level had a decreased trend with the increase of fermented HTR replacement, and we therefore inferred that fermented HTR may be required to alleviate the cell damage caused by heat stress. T-AOC level increased significantly in 15% substitution and SOD level increased significantly in 30% substitution group, reflecting the antioxidant activity of fermented HTR, which was consistent with Chaudhary’s findings [47].

In addition, some studies have suggested that to adapt to heat stress, the body needs to increase the absorption of glucose to ensure the energy supply, resulting in an increased serum glucose content [48]. In the present study, there was no significant difference in the total cholesterol level with increasing fermented HTR. However, under heat stress, the glucose level of the fattening cattle fed with the basal diet was significantly higher than that of cattle fed with the 30% fermented HTR alternative diet, suggesting that heat stress has an effect on the glucose level, which can be alleviated by fermented HTR. Compared with those of the CN group, the total protein and albumin of the fermented HTR groups increased while blood urea nitrogen showed a decreased trend, which was similar with Guo’s results [49]. Guo pointed out that this might be the result of thyroid regulation, which was conducive to maintaining the body’s water and salt balance, and alleviating the adverse effects of heat stress [49].

At present, there are few studies on the effect of HTR on the fecal microorganisms of fattening cattle under heat stress. Recently, a study showed that there are significant differences in the microbial composition between spring and summer, indicating that environmental temperature has an impact on the microbial community structure of feces [50]. In the present study, we found that 30% fermented HTR had a more significant effect on heat stress index of fattening cattle than 15%; therefore, we chose the CN and HC group to study the fecal microbial community of fattening cattle. According to the results of alpha diversity analysis and PCA, the fecal microbial community of heat stressed fattening cattle fed with fermented HTR was significantly different from cattle fed with the basic diet, and the microbial diversity of the HC group increased. The most abundant bacteria in the phylum level of the CN and HC group were *Firmicutes* and *Bacteroidetes*. Li showed that under the different climates in spring and summer, the main microorganisms in cow feces were *Firmicutes* and *Bacteroidetes*; in summer, *Firmicutes* levels were higher than those in spring, while *Bacteroidetes* levels were lower than those in spring [50]. It has been reported that ruminants have a core microbial community mainly composed of *Firmicutes* and *Bacteroidetes*, which can maintain stable digestive function [51]. In our study, the feeding of fermented HTR changed the relative proportion of these two bacteria, which was consistent with Hu’s study [52]. The results showed that the enrichment of *Firmicutes* in the HC group was significantly higher than that in CN group, and *Bacteroidetes* levels in HC group were significantly lower, indicating that fermented HTR had a certain effect on fecal microbial flora of fattening cattle under summer heat stress. It is reported that *Bacteroidetes* is mainly responsible for the degradation of fiber, and its degree of enrichment is related to diets with a high fiber content. By contrast, *Firmicutes* are responsible for the degradation of carbohydrates, fats, and proteins, which are related to a high calorie diet [53]. We infer that the increase of *Firmicutes* in HC group is related to the richer crude protein and crude fat in fermented HTR.

At the family taxonomic level, the *Rikenellaceae* and *Paludibacteraceae* in the HC group decreased, and the *Ruminococcaceae* increased. It has been reported that the *Rikenellaceae* increased and the *Ruminococcaceae* decreased in the intestines of mice fed with a high-fat diet, suggesting that a high calorie diet can cause changes in the intestinal microflora [54], which is contrary to the results of the HC group and might be explained by the fact that under heat stress, the fermented HTRs reduced the caloric level of the diet, alleviated the heat production during fattening, and changed the intestinal flora. At the genus taxonomic level, *Fournierella* and *Oscillibacter* are two genera of the *Ruminococcaceae*, and both showed an increasing trend in the HC group. *Acetobacterium*, *Anaerovorax*, *Butyrivibrio*, and *Erysipelotrichaceae* also increased in the HC group. A study has reported that *Butyrivibrio* is more involved in epithelium promotion, while *Erysipelotrichaceae* is more abundant at high temperatures [55]. Besides, *Alistipes* decreased in the HC group. A study suggested that *Alistipes* is the source of colorectal cancer, and its abundance is related to the severity of intestinal diseases [56], which indicates that fermented HTR is beneficial to maintain the health of fattening cattle under heat stress.

We also studied the association between fecal SCFAs and microorganisms at the genus level where changes were observed. We found that there was a significant positive correlation between Aa and the relative abundance of *Acetobacterium* and a positive correlation between Pa and the relative abundance of *Acetobacterium*, *Erysipelotrichaceae*, and *Fournierella*. It has been reported that *Acetobacter* is mainly involved in Aa fermentation [57] and *Fournierella* can metabolize and produce Aa and Pa [58]. SCFAs are the main products of microbial metabolism in the intestines, and the types and quantity of SCFAs are regulated by microbial species, diet, and the environment [23]. Therefore, we speculated that fermented HTRs change the composition and abundance of large intestine microorganisms in fattening cattle, promote the fermentation of large intestine microorganisms, and help the body to maintain health and resist the adverse effects of heat stress.

## Conclusions

After fermentation with oat hay and compound bacteria, the pH of the HTR decreased and the nutritional level increased, which was conducive to the resource utilization of HTR. In addition, fermented HTR could alleviate the heat stress of fattening cattle in summer, improve their growth performance, enhance their immune performance, help them to resist an adverse environment, and maintain their health. In addition, 30% fermented HTR replacement had an impact on the fecal microbial community of heat stressed fattening cattle, and the bacteria were closely related to the cattle’s fecal SCFA levels. Based on our findings, we concluded that 30% fermented HTRs replacing *Pennisetum purpureum* grass might be advantageous for fattening cattle under heat stress. In addition, the co-fermented products also included molasses, oat hay, and mixed bacterial strains in our test, and the potential roles of these co-fermented products on heat stress should be further study.

## Materials and methods

### Fermentation

The HTRs used in the experiment were obtained from Heyuan Jilongxiang Biological Technology Co., LTD. (Heyuan, Guangdong, China). The molasses were purchased from Guangzhou Linong Feed Co., Ltd. (Guangzhou, Guangdong, China) and strains were purchased from Guangdong Microbial Culture Collection Center (Guangzhou, Guangdong, China). The feedstock department of Guangzhou Fengxing Dairy Co., LTD. (Guangzhou, Guangdong, China) provided the oat hay.

Based on dry matter, the tested HTR contained 20% Mesona chinensis leaves, 20% *Plumeria rubra* flowers, 20% Microcos paniculata leaves, 12% Honeysuckle flowers, 12% Chrysanthemum flowers, 12% *Prunella vulgaris* leaves and 4% Liquiritia glycyrrhiza roots in mass ratios, respectively. The nutritional components of HTR was analyzed by E-more Technology Co., Ltd. (Ulanqab, Inner Mongolia, China). In details, HTR was grind to power in 1 mil with UDY-cyclone (UDY Corporation, Fort Collins, CO); then scanned by NIR spectroscopy with ‘Feed and Forage’ system (1100-2500 nm) (Thermo Scientific, Waltham, Massachusetts, US) and merged with Dairy on (https://dairyone.com/) database.

In this experiment, HTR and oat hay were cut into 2–3 cm pieces. Fermentation was carried out on HTR and oat hay (CP: 12.3%; NDF: 50.8%; ADF: 26.7%) with a mass ratio of 6:4 on a wet weight basis (approximately 3:7 on dry matter basis), with 2% added molasses. The mixed bacteria used for fermentation were *Saccharomyces cerevisiae GDMCC2.167* (3 × 10^8^ colony forming units/g), *Bacillus subtilis GDMCC1.372* (3 × 10^8^ colony forming units/g), *Lactobacillus plantarum GIM1.191* (5 × 10^9^ colony forming units/g) and *Enterococcus faecalis GDMCC1.612* (3 × 10^8^ colony forming units/g), with mass ratios of 250 g: 1 t, 300 g: 1 t, 300 g: 1 t, and 200 g: 1 t, respectively. The fermentation materials were mixed and stirred evenly using a feed-stuff mixer. Then the mixture was compacted to remove air and pressed into polyethylene bags (50 kg each) and sealed, and fermented for 25-30 days at room temperature. After approximately 30 days of fermentation, 20 g of fermented samples were added to 180 mL of distilled water, sealed with preservative film, and placed in a refrigerator at 4 °C for 24 h. During extraction, the solution was shaken every 30 min. After 24 h, the solution was filtered through qualitative filter paper, and a pH meter (Horiba, Tokyo, Japan) was used to determine the pH value of the filtrate. High performance liquid chromatography (HPLC) was used to determine the content of organic acids in the extract of fermented HTRs, while AOAC International guidelines [59] were used to analyze the dry matter, crude protein, NDF, ADF, and ash contents.

### Experimental animals and design

This experiment was carried out in Guangzhou from July to August in 2019, and the weather is hot, rainy and humid, which usually leads to a natural heat stress and we judged whether the individuals were in the state of heat stress by using the environmental temperature and humidity index [22]. A total of 45 healthy female Simmental crossbred cattle (18 months old), balanced with body weight, were selected and divided into three treatment groups (15 cattle per group) in the same cowshed: The CN group (fed with basal diet, without fermented HTR), the LC group (fermented HTR replaced 15% of *Pennisetum purpureum* grass), and the HC group (fermented HTR replaced 30% of *Pennisetum purpureum* grass). The experiment lasted for 40 days, including a 7-day adaptation period and a 33-day formal experimental period.

The animal feeding experiment was carried out at the Yunfu benben beef cattle farm (Yunfu, Guangdong, China), and the basal diet for the experimental cattle was provided by the farm and was shown in Table S2. During the experiment, all individuals were fed at 8:00 am and 5:00 pm every day. A total mixed ration (TMR) feed mixer was used to fully mix the experimental diets, and the diet and drinking water were allowed to intake freely during the experiment.

### Measurements and sampling

During the experiment, a dry and wet thermometer (Kimo Industry Co., Biarritz, France) was set at 1.5 m above the center of ground in the cowshed. The temperature, humidity, and wet bulb temperature of the cowshed were recorded at 08:00, 14:00, and 20:00 every day to calculate the daily average temperature, humidity and the temperature humidity index (THI) of the cowshed. The following formula was used to calculate the THI: THI = (0.35 × TDB + 0.65 × TWB) × 1.8 + 32, where TDB is the dry bulb temperature and TWB is the wet bulb temperature [22].

In every 3 days, an animal thermometer (Kruuse, Langeskov, Denmark) was used to measure the rectal temperature (RT) of the experimental cattle at 08:00, 14:00, and 22:00 during the formal experimental period. When the tested cattle lay still, we counted the times of chest fluctuation in 1 min with the counter, which was recorded as respiratory rate (RR). At the beginning and end of the formal experiment, the weights of the experimental cattle were recorded after a 12-h overnight fast. The amount of TMR consumed by the experimental cattle, and that remaining unconsumed, were recorded every day. The above data were used to calculate the average daily feed intake (ADFI), average daily gain (ADG), and the feed to weight ratio (F/G). On the day before the end of the experiment, six individuals in each treatment group were randomly selected and 20 mL blood samples were taken from the tail vein, placed in a centrifuge tube, and then centrifuged immediately after being placed obliquely for 2 h. Enzyme linked immunosorbent assay (ELISA) kits were used to determine the serum levels of heat shock protein 70 (HSP70), cortisol (Cor), lactate dehydrogenase (LDH), immunoglobulin G (IgG), immunoglobulin A (IgA), alanine aminotransferase (ALT), creatine kinase (CK), total antioxidant capacity (T-AOC), malondialdehyde (MDA), superoxide dismutase (SOD), glutathione peroxidase (GSH-PX) according to the manufacturer instrutions (Nanjing Jiancheng Bioengineering Research Institute, Nanjing, China).

At the end of the experiment, six animals of each group were randomly selected and 200 g feces were collected from 12 fattening cattle rectums in CN and HC groups, then put into a frozen storage tube and stored in liquid nitrogen. Approximately 100 g were used for HPLC (Actlabs, Ancaster, ON, Canada) determination of fecal volatile fatty acid contents, including acetic acid (Aa), propionic acid (Pa), isobutyric acid (Ia), butyric acid (Ba), isovaleric acid (Iva), and valeric acid (Va). The other 100 g were used to extract total genomic DNA to investigate fecal microorganisms and the total genomic DNA was extracted using CTAB/SDS method. DNA concentration and purity was monitored on 1% agarose gels. According to the concentration, DNA was diluted to 1 ng/μL using sterile water.

### 16S rRNA gene sequencing and annotation analysis

We amplified the V3 and V4 regions of the 16S rRNA sequence of the bovine fecal microbial communities by using a forward primer (5′-GTGCCAGCMGCCGCGG-3′) and a reverse primer (5′-GGACTACHVGGGTWTCTAAT-3′), and then constructed gene fragment libraries. The paired end method was used to sequence the libraries on the Illumina Novaseq sequencing platform (Illumina, Waltham, MA, USA). High-quality tags data (clean tags) were obtained after splicing and filtering of the reads [60], and then mosaic filtering was performed to obtain effective tags for subsequent analysis. The Uparse software (Uparse v7.0.1001) [61] was used to cluster all effective tags of all samples. Sequences with ≥97% identity were clustered into same operational taxonomic units (OTUs). Then, Silva databases and Mothur [62] were used to annnotate and analyze the OTU sequences. The composition and abundance of microbial communities in each sample were counted at each taxonomic level, including kingdom, phylum, class, order, family, genus, and species. Fast multiple sequence alignment and annotation was carried out using the MUSCLE software (Version 3.8.31), and the phylogenetic relationships between different OTUs were obtained [63]. Finally, the data of each sample were homogenized, and the subsequent Alpha and Beta diversity were analyzed based on the homogenized data. The QIIME pipeline (Version 1.7.0) was used to calculate the UniFrac distance and an Unweighted Pair-group Method with Arithmetic Means (UPGMA) clustering tree was also constructed. The R software (Version 2.15.3) was used to draw Principal Component Analysis (PCA), Principal Co-ordinates Analysis (PCoA), and Non-Metric Multi-Dimensional Scaling (NMDS) maps to explore the differences of community structure among the different samples. The Linear discriminant analysis Effect Size (LEfSe) analysis was performed with a linear discriminant analysis LDA score of 2 to further analyze the differences in community structure.

### Statistical analysis

One-way analysis of variance (ANOVA) and Duncan’s test (SPSS 17.0, IBM Corp., Armonk, NY, USA) were used to analyze the physiological parameters, serum biochemical indices, and the fecal concentrations of short-chain fatty acids (SCFAs). The results are shown as the mean ± standard error (SE), and *P* < 0.05 and *P* < 0.01 were used as the criteria to judge significant and extremely significant differences.

## Supplementary Information

**Additional file 1 **: **Figure S1.** Daily changes in cowshed ambient temperature and humidity index (THI) in three time. The red line represents the daily changes of THI index at 8:00 am, the orange line represents the daily changes of THI index at 15:00 pm, and the gray line represents the daily changes of THI index at 22:00 pm. **Supplementary Tables S1-S6**.

## Data Availability

The raw sequences were deposited into Sequence Read Archive (SRA) database with the BioProject accession number PRJNA699165.
